# Body Composition, Bone Health, and Dietary Intake in Children After Allogeneic Hematopoietic Stem Cell Transplantation

**DOI:** 10.3390/nu18132193

**Published:** 2026-07-06

**Authors:** Janne Anita Kvammen, Rut Anne Thomassen, Kristin Godang, Jochen Buechner, Jens Bollerslev, Beint Sigmund Bentsen, Anne Grete Bechensteen, Christine Henriksen

**Affiliations:** 1 Department of Pediatric Medicine, Oslo University Hospital, 0424 Oslo, Norway; 2 Department of Nutrition, Institute of Basic Medical Sciences, Faculty of Medicine, University of Oslo, 0317 Oslo, Norway; 3 Section of Specialized Endocrinology, Department of Endocrinology, Oslo University Hospital, 0424 Oslo, Norway; 4 Department of Pediatric Hematology and Oncology, Oslo University Hospital, 0424 Oslo, Norway; 5 Faculty of Medicine, University of Oslo, 0372 Oslo, Norway

**Keywords:** body composition, bone mineral density, nutrition, nutritional status

## Abstract

**Background/Objectives:** This study describes body composition, bone mineral density (BMD), and dietary intake in pediatric patients undergoing allogeneic hematopoietic stem cell transplantation compared to healthy children. **Methods:** In this prospective observational study, dual-energy X-ray absorptiometry was used to assess appendicular lean mass index (ALMI), fat mass index (FMI), fat mass percentage (FM%), and BMD, and a 4-day dietary record was used to assess dietary intake at 3 months and 1 year post-transplant. Healthy children were assessed once by the same methods. **Results:** We included 28 patients (mean 10.3 years, SD 4.0) and 50 healthy children (mean 10.0 years, SD 3.6). At 1 year, median Z-scores were lower for ALMI (−1.34 vs. 0.40, *p* < 0.001), higher for FMI (0.34 vs. −0.33, *p* < 0.012) and FM% (0.59 vs. −0.98, *p* < 0.001), lower for BMD total body less head (−1.0 vs. 0.3, *p* = 0.006), but similar for BMD spine compared to healthy children. At 1 year, 9/15 (60%) had ALMI Z-score ≤ −1, 6/15 (40%) had FMI Z-score ≥ 1, 5/15 (33%) had FM% Z-score ≥ 1, and 8/18 (53%) had BMD total body less head Z-score ≤ −1, and 3/15 (20%) had BMD spine Z-score ≤ −1. Dietary intake improved, and at 1 year, energy and protein intakes were comparable, fat, calcium, and vitamin D intakes were higher, but fiber intake remained lower in patients than in healthy children. **Conclusions**: Patients had a more unfavorable body composition and bone health. Dietary intake improved from 3 months to 1 year post-transplant. However, the results indicate a need for nutritional follow-up, particularly targeting protein, fat, fiber, calcium, and vitamin D.

## 1. Introduction

Allogeneic hematopoietic stem cell transplantation (HSCT) can potentially cure children with life-threatening malignant, immunological, hematological, and metabolic diseases [1]. However, survivors are at risk of chronic health complications that negatively impact quality of life [2]. Ensuring healthy nutrition and maintaining physical activity could prevent malnutrition and delay the onset of chronic diseases, such as sarcopenia, osteoporosis, cardiovascular diseases, and secondary cancers [2,3,4,5,6,7,8]. Therefore, optimal nutrition may be even more critical for HSCT survivors than for the general population. However, studies of childhood cancer survivors show poor dietary habits and insufficient intake of micronutrients [4,9,10].

Growth deficits, unfavorable body composition, impaired bone health and metabolic vulnerability after HSCT and childhood cancer treatment share several common denominators: the underlying disease, aggressive multi-modal therapies (e.g., methotrexate and corticosteroids, total body irradiation), treatment-related complications (e.g., growth hormone deficiency, graft-versus-host disease, infections), altered metabolism, unbalanced diet, malnutrition, micronutrient deficiencies and inactivity [2,7,11,12,13,14].

In HSCT and childhood cancer survivors, an unfavorable body composition characterized by low muscle and increased fat mass has been detected early in the treatment course [15,16,17]. A recent study observed that young pediatric cancer survivors with sarcopenia had a higher risk of low bone mineral density (BMD) [18], reflecting the strong interplay between muscle and bone mass [19]. Furthermore, decreased BMD Z-scores have been documented in HSCT and childhood cancer survivors [7,20]. Low BMD is an important risk factor for osteoporosis and fractures, underscoring the importance of optimizing peak bone mass during adolescence [19].

We have previously shown that a combination of enteral and parenteral nutrition ensured stable weight in this study cohort during the first month after transplantation [21]. However, body composition, BMD, and nutritional intake have not been studied simultaneously during the first year after HSCT. Early identification of unfavorable body composition and decreased BMD, combined with dietary assessment, is essential to target lifestyle interventions. Therefore, we aimed to evaluate these factors in children undergoing HSCT at 3 months and 1 year post-transplant and compare results with those of healthy children. Our specific objectives were to describe (i) anthropometric measures, (ii) body composition, (iii) BMD, and (iv) nutritional intake.

## 2. Materials and Methods

### 2.1. Ethics Statement

Informed consent was obtained from all parents and adolescents ≥16 years. The ethical standards of the Declaration of Helsinki were followed. The Regional Ethics Committee South-East (2016/391/REK sør-øst B) and the Data Protection Officer, OUH, approved the study. The study was registered in ClinicalTrials.gov (Trial registration number: AEV2017/1 (NCT03049826), Registration date: 10 February 2017). Generative artificial intelligence has not been used.

### 2.2. Design and Subjects

In this prospective observational study, we included 2–18-year-old children undergoing HSCT at the Department of Pediatric Hematology and Oncology, Oslo University Hospital, Norway. Patients were assessed at hospital admission before conditioning therapy (baseline), at the 3-months and 1-year follow-ups after transplantation. Data collection was conducted from April 2018 until January 2022. According to hospital practice, a registered dietitian provided individualized dietary advice during hospitalization for HSCT, at the 3-months and 1-year follow-ups. From March through September 2017, at the Centre for Clinical Nutrition, University of Oslo, we conducted a cross-sectional assessment of a convenience sample of 50 healthy children recruited via social media. Details on recruitment and inclusion criteria have been previously published [21,22].

### 2.3. Anthropometry

Standardized procedures were used to assess anthropometrics. A Seca weight (model 7701321004, Seca GmbH & Co. KG, Hamburg, Germany) was used to measure weight (kg), and a stadiometer (Holtain Limited, Pembrokeshire, Wales, UK) was used to measure height (cm) in patients. Healthy children were measured by a combined digital Seca 284 (Seca GmbH & Co. KG, Hamburg, Germany). Growth was assessed by Z-scores based on Norwegian growth references [23]. We categorized participants as stunted if their height Z-score was <−2, thin if their BMI Z-score was <−2, and overweight (including obese) if their BMI Z-score was >1 [24].

### 2.4. Dual-Energy X-Ray Absorptiometry

Dual-energy X-ray absorptiometry (DXA), the gold standard for assessing body composition and bone mass, is rapid, precise, and considered safe, with a very low effective X-ray dose [25]. Patients were measured at their 3-months and 1-year follow-ups at the Department of Specialized Endocrinology, Oslo University Hospital. Lunar Prodigy (Lunar, GE Healthcare, Corp., Madison, WI, USA) was used until September 2021, and Lunar iDXA afterwards due to a change of equipment. Healthy children were measured once using Lunar iDXA at the University of Oslo. Daily calibration procedures for bone and fat were performed to avoid systematic errors across machines. We measured body composition (fat mass, lean mass), bone mass, and body areas (cm^2^) in participants aged five years or older.

Bone mineral density (BMD (g/cm^2^) for the anterior-posterior lumbar L1–L4 spine and total body less head were assessed by the International Society of Clinical Densitometry (ISCD) Official Positions [25]. Software enCORE version 16 (GE Healthcare, Corp., Madison, WI, USA) with age, gender, and ethnicity-appropriate reference materials from the National Health and Nutrition Examination Survey (NHANES)/Lunar (combined NHANES/Lunar) was used [26,27]. Corrected age was used to evaluate DXA results for five patients, where hand radiographs verified delayed skeletal growth [28]. BMD Z-scores from −1 to >−2 were defined as low, and ≤−2 as very low. A decreased BMD Z-score was defined as a combination of low and very low BMD Z-score [7,25].

Appendicular lean mass (ALM, kg) is the sum of the lean mass of arms and legs, and was used to assess muscle mass. ALM index (ALMI, kg/m^2^) and fat mass index (FMI, kg/m^2^) were calculated by dividing the mass (kg) by the square of the height (m^2^). Percentage fat mass (FM%) was calculated as FM (kg) divided by total body weight (kg). Gender and age-appropriate European references from the Austrian LEAD study were used to calculate Z-scores for ALMI and FMI (LEAD calculators—LEAD Study (https://leadstudy.at/lead-cva-calculator/)/Z-Score App (https://floriankrach.github.io/pyscripts/zscore-app-pyscript/index.html) (URLs accessed on 5 September 2024) for participants ≥ 6 years [29]. Low ALMI were defined as Z-score −1 to >−2, and very low ALMI as Z-score ≤ −2. A decreased ALMI Z-score was defined as a combination of low and very low ALMI. High FMI and FM% were defined as Z-scores ≥ 1.

### 2.5. Dietary Assessment

A 4-day food record was completed at the 3-months and 1-year follow-ups in patients and once in healthy children. All foods, liquids, tube feeds, parenteral nutrition, and dietary supplements were registered [30]. Household measures, supported by a booklet with pictures of typical dishes and portion sizes, were used to improve dietary reporting [31]. Participants noted receipts on homemade foods, including cooking methods, which were incorporated into the dietary analyses. In patients using parenteral nutrition, the mean daily energy and nutrient intakes were calculated from the weekly provision based on information from medical charts and hospital pharmacy prescriptions. Total intakes of energy, macronutrients, and selected micronutrients were determined by using a nutritional analysis software (Dietist Net Pro version 2) based on the Norwegian Food Composition Table [32], and compared to the daily recommended intake from the Nordic Nutrition Recommendations 2023 (RI) [33].

### 2.6. Statistics

Statistical analyses were performed using IBM SPSS Statistics for Windows, version 29 (Armonk, New York, NY, USA). Normally distributed data were presented as means and standard deviations (SD), and non-normally distributed data as medians with interquartile ranges (IQR). Absolute numbers and percentages were used to evaluate the prevalence of participants with malnutrition, impaired body composition or BMD, and dietary intake below recommendations. To assess pairwise differences, we used an independent samples *t*-test, Mann–Whitney U test, paired samples *t*-test, and Wilcoxon Signed-Rank test, as appropriate. The Chi-square test or Fisher’s Exact test was used to assess and explore the differences in categorical variables. Only complete case analyses were carried out, that is, no imputation of missing data. The significance level was two-sided and set to *p* < 0.05.

## 3. Results

### 3.1. Characteristics of Participants

Participant characteristics are presented in Table 1. The baseline characteristics have been published previously [21,22]. In short, both groups had a mean age of 10 years. We included 28 patients aged 3.5 to 16.6 years and 50 healthy children aged 3.6 to 16.8 years. Four patients died during the first year post-transplant, leaving 26 patients for assessment at the 3-month and 24 patients at the 1-year follow-up. There were more boys in the HSCT group than in the healthy children group. Most patients, 18/28 (64%), were treated for malignant conditions. Myeloablative conditioning therapy was predominantly used, and seven participants with acute lymphoblastic leukemia received total body irradiation. Bone marrow from a matched unrelated donor was used in more than two-thirds.

At all three time points, patients were shorter, but had similar BMI Z-scores compared to healthy children. Thinness was not detected, but overweight was found in 7/28 (25%) at baseline, and in 4/24 (17%) at 1 year. Stunting was observed in 5/28 (18%) at baseline and 5/24 (21%) at 1 year (Table 1). Within patients, Z-scores for weight (*p* = 0.029) and height (*p* = 0.014) were lower at 3 months than at baseline. However, at 1 year, no significant differences from baseline were detected.

### 3.2. Body Composition and Bone Health

Valid DXA measurements were available for 18/26 (69%) of patients at 3-month, 15/24 (63%) at 1-year follow-up, and in 46/50 (92%) healthy children. Age or gender did not differ between patients who were assessed with DXA and those who were not assessed (at 1 year, *p* = 0.128 and *p* = 1.000). Among patients who were not measured at both time points, one missed measurement was due to the COVID-19 pandemic, one to adverse weather conditions, and one to logistical issues at the hospital. Additional reasons for not undergoing DXA at 1-year follow-up were: three participants were younger than five years, two declined DXA, and one further case was related to the COVID-19 pandemic. Among healthy children not assessed by DXA, three participants were younger than five years and one could not manage the procedure. As well, two healthy participants did not measure the spine, leaving 44/50 (88%) DXA results for L2–L4.

**Figure 1:**
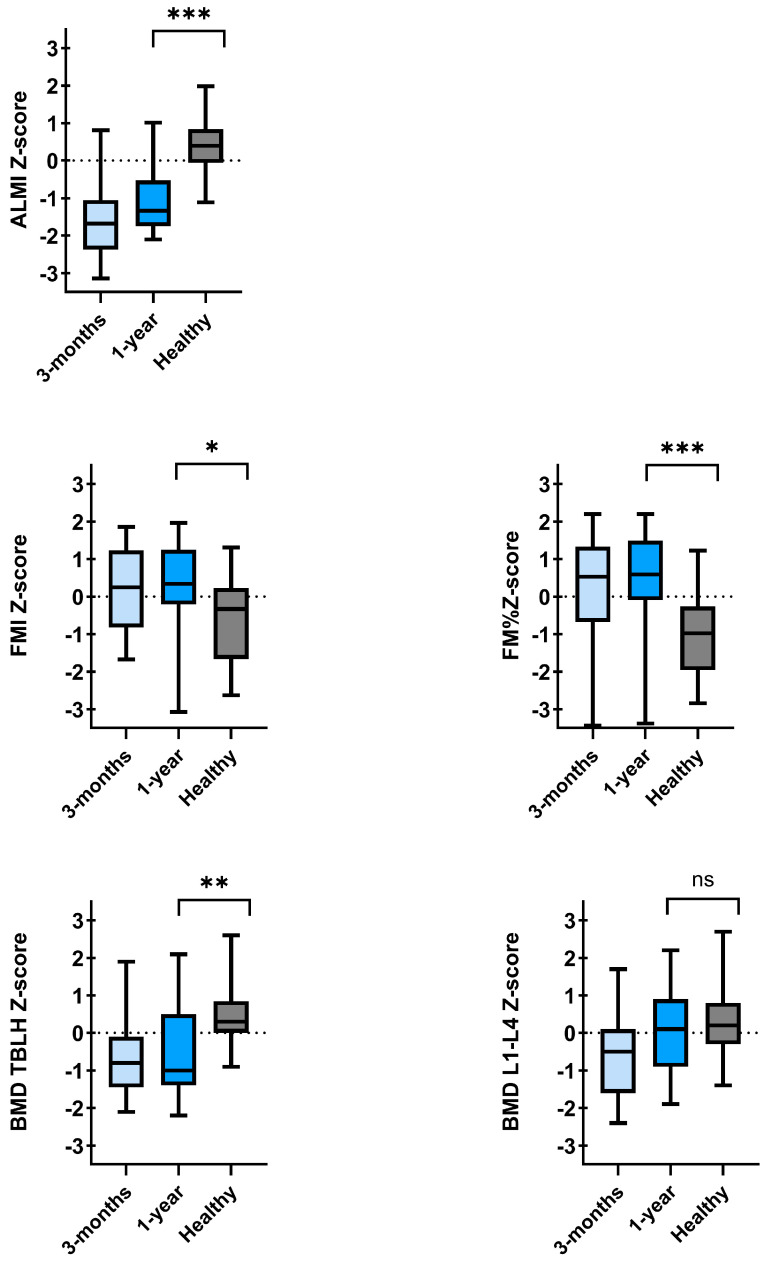
Body composition and bone mineral density at 3-month and 1-year post-transplant follow-up in children undergoing HSCT and in healthy children. Abbreviations: ALMI = appendicular lean mass index, BMD = bone mineral density, FM% = fat mass percentage, FMI = fat mass index, HSCT = hematopoietic stem cell transplantation, L1–L4 = spine lumbar vertebrae L1–L4, TBLH = total body less head. For ALMI, FMI, and FM% Z-scores: *n* = 16 at 3 months, *n* = 15 at 1 year, and *n* = 42 for healthy children. For BMD: *n* = 18 at 3 months, *n* = 15 at 1 year, and BMD TBLH: *n* = 46 and BMD L2–L4: *n* = 44 for healthy children. Statistical differences between HSCT at 1-year follow-up and healthy children indicated: ns = non-significant, * = *p* < 0.05, ** = *p* < 0.01, *** = *p* < 0.001.

Z-scores for ALMI, FMI and FM% were calculated for 16/26 (62%) at 3 months, 15/24 (63%) at 1 year and in 42/50 (84%) of healthy children. Figure 1 and Table A1 present median Z-scores for body composition and BMD results. Compared to healthy children, median ALMI Z-scores were lower and FMI and FM% Z-scores higher at both 3 months and 1 year, indicating a more unfavorable body composition with lower muscle mass and higher fat mass. Compared to healthy children, BMD total body less head Z-scores were lower at both time points. Spine BMD Z-scores were lower at the 3-month, but similar to healthy children at the 1-year follow-up.

**Figure 2:**
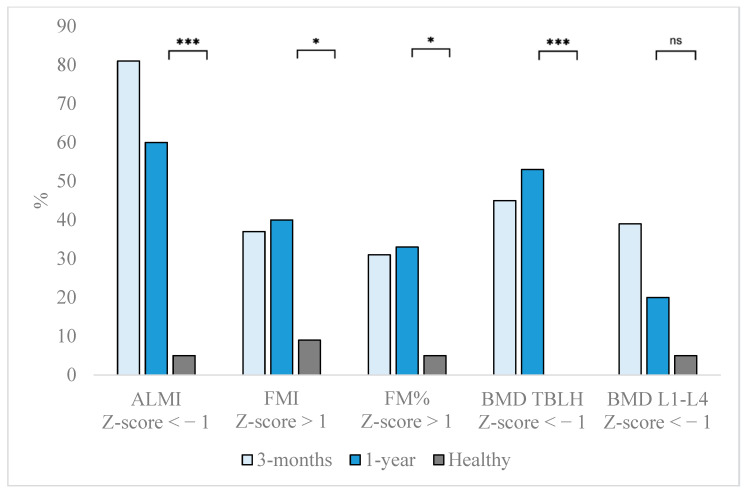
Decreased ALMI Z-scores, high FMI Z-scores, high FM%, and decreased BMD Z-scores at 3-month and 1-year post-transplant follow-up in children undergoing HSCT and in healthy children. Abbreviations: ALMI = appendicular lean mass index, BMD = bone mineral density, FM% = fat mass percentage, FMI = fat mass index, L1–L4 = spine lumbar vertebrae L1–L4, TBLH = total body less head. For ALMI, FMI, and FM%: *n* = 16 at 3 months, *n* = 15 at 1 year, and *n* = 42 for healthy children. For BMD: *n* = 18 at 3 months, *n* = 15 at 1 year, and BMD TBLH: *n* = 46 and BMD L2–L4: *n* = 44 for healthy children. Statistical differences between HSCT at 1-year follow-up and healthy children indicated: ns = non-significant, * = *p* < 0.05, *** = *p* < 0.001.

Figure 2 and Table A2 present the prevalences of different categories of body composition markers and BMD results. Higher prevalences of decreased ALMI, high FMI, high FM%, and decreased BMD total body less head Z-scores were detected in patients compared to healthy children.

### 3.3. Dietary Intake

Diet records were available for 23/26 (89%) of patients at 3 months and 18/24 (75%) at 1 year. Post-transplant, the median duration of tube feeding was 37 days (9–169), and the median duration of parenteral nutrition was 37 days (28–54). At 3 months, four patients received supportive parenteral nutrition, which accounted for 42–71% of their total energy intake. Eight patients received some supportive tube feeding, and three used variable amounts of oral nutrition support products. In total, half of the patients received some nutritional support. At 1 year, none had parenteral nutrition support, 4/18 (22%) used some supportive tube feeding (Table A3).

Table 2 presents daily provision of energy, macronutrients and selected micronutrients in patients at 3-month and 1-year post-transplant follow-up compared to healthy children. Patients had lower intake of energy (kcal), protein (g and g/kg), carbohydrate (g), fiber (g and g/MJ), and calcium (mg) at 3 months compared to healthy children. In contrast, intake of fat (g) and sugar (g) did not differ between groups. Energy-% from protein was lower among patients, whereas energy-% from carbohydrate, fat, and sugar did not differ from healthy children.

At 1 year, energy (kcal), protein (g and g/kg), carbohydrate (g), and sugar (g) intakes in patients were similar to those of healthy children. Intake of fat (g), calcium (mg), and vitamin D (µg) (as previously published [22]) was higher, while fiber (g and g/MJ) remained lower compared to healthy children.

Compared to the RI [33], insufficient calcium intake was frequently detected in both groups. Vitamin D intake below RI was less frequent among patients than among healthy children.

As shown in Table A3, patients receiving nutritional support were younger than those on a regular diet at both 3 months and 1 year after HSCT. Energy percentages from macronutrients did not differ between patients receiving nutritional support and those on a regular diet.

## 4. Discussion

The main finding of this study was that children during the first year after HSCT exhibited a more unfavorable body composition, characterized by lower muscle mass, higher fat mass, and lower BMD, despite similar BMI when compared to healthy children. Overall dietary intake was lower at the 3-month, but at the 1-year follow-up, energy and protein intakes had increased, and calcium and vitamin D intakes were higher than those of healthy children. However, higher fat and lower fiber intake among patients at 1-year follow-up were concerning.

Patients had shorter stature than healthy children. One in five was stunted, reflecting the impact of chronic illness [24]. However, height Z-scores did not change from baseline to 1-year follow-up. Our results support previous reports of impaired growth in 9–85% of HSCT survivors [2,34]. A recent study showed that prepubertal children were at higher risk of stunted growth 12–15 years post-transplant compared to those transplanted during puberty [35]. The wide variation reflects studies of heterogeneous patient groups across diagnoses, medical therapy, age, and follow-up duration.

In our study, the prevalence of overweight was stable from baseline until 1 year post-transplant. Risk of rising BMI up to five years post-transplant has previously been demonstrated [36]. Importantly, maintaining a healthy lifestyle, including a normal BMI, was associated with a 20–30% reduction in mortality 40 years or more after being diagnosed with childhood cancer [37]. Our findings might indicate a window of opportunity for early prevention of developing overweight.

Our patients had lower muscle mass and higher fat mass than healthy children, despite similar BMI. Previously, a profound loss of lean body mass from hospitalization until 3 months after HSCT has been shown [17]. A body composition with low lean mass and increased fat mass has also been reported in other studies of HSCT survivors [11,38] and in childhood cancer survivors [39,40]. These results reflect the burden of illness, treatment, and medication side effects, metabolic alterations, as well as possible effects of low activity and nutritional intake [41]. Reduced insulin sensitivity and dyslipidemia have also been found in HSCT survivors [38], indicating an increased cardiovascular risk, which potentially is linked to this unfavorable body composition phenotype. Furthermore, loss of lean mass is concerning because it has been associated with length of hospitalization, morbidity, and mortality [24].

Compared with healthy children, we observed lower BMD Z-scores at both the 3-month and 1-year follow-ups. The risk of impaired bone health is consistent with previous reports of both short and long-term survivors, with higher rates in patients with complications, such as chronic graft-versus-host disease [38,42]. Findings suggest that decreased BMD was more prevalent among patients transplanted during puberty than those transplanted at younger ages [35], most likely reflecting increased vulnerability during the period of most rapid bone accretion [19]. A recent meta-analysis confirmed an increased risk of reduced BMD in childhood cancer survivors compared to controls [20]. High prevalence of osteoporosis has also been shown among survivors of adult HSCT [43]. Risk of impaired bone health is supported by evidence of increased bone resorption [44] and suppressed bone formation after HSCT [42], suggesting a direct adverse impact on bone metabolism. Furthermore, a recent study of pediatric cancer survivors reported significant associations between sarcopenia and low BMD Z-score 5 years after the completion of cancer therapy [18], underscoring the close crosstalk between muscle and bone tissue [19].

Taken together, body composition and bone health after HSCT may be influenced by multiple factors, including total body irradiation, graft-versus-host disease, corticosteroid use, endocrine alterations, nutritional factors, and physical activity [2]. Findings highlight the importance of offering young survivors lifestyle advice and interventions to optimize musculoskeletal health. Peak bone mass has not yet been reached, and restoration of musculoskeletal health might be possible.

This study has previously described nutrient intake during the first four weeks post-transplant [21], and here we show that intake improves over the first year. At 3 months after transplantation, patients had lower energy, protein, fiber, and calcium intake than healthy children, reflecting dietary challenges following transplantation, such as gastrointestinal dysfunction and taste changes [45]. At 1 year, energy and protein intake were similar to that of healthy peers, most likely reflecting improved health and increased physical activity. The optimal protein requirement at different time points during the transplant course remains unknown [46]. A recent systematic review found no studies on the effects of protein supplementation interventions on anthropometric measures, body composition, physical fitness, or adverse effects in pediatric oncology [47]. Nevertheless, proteins are essential for muscle mass [48], and further research is warranted.

Higher fat and lower fiber intake in patients compared to healthy children is concerning. Poor dietary patterns, also with low intake of fruits and vegetables, in childhood cancer survivors have been demonstrated in several studies [4]. A high-fat diet could increase the risk of becoming overweight, developing an unfavorable body composition, and contributing to the risk of cardiovascular disease. Furthermore, dietary fiber is crucial for gut microbiota rehabilitation [49] and may also reduce the risk of chronic graft-versus-host disease [50]. Higher microbiota diversity has been associated with lower mortality in adult HSCT patients [51]. Dietary advice emphasizing a healthy diet seems important in the follow-up of HSCT patients.

Our patients had a lower calcium intake at the 3-month but higher than healthy children at the 1-year follow-up. At both time points, patients had higher vitamin D intakes, which were related to individual supplementation based on vitamin D status measurements, as previously reported [22]. These results most likely reflect targeted dietary advice. Inadequate dietary intake of several micronutrients, including calcium and vitamin D, has also been observed among long-term survivors of pediatric HSCT [52]. In general, lifestyle choices account for 20–40% of peak bone mass, and the positive effects of adequate calcium intake, physical activity, vitamin D, and dairy products on bone health have been documented [19].

The main strengths of this study were its prospective design and comparison of results with those of healthy children. Results improve our understanding of body composition, bone health, and dietary intake after HSCT in children. New information on dietary intake could be used in clinical practice to improve dietary follow-up during the first year after HSCT. Evaluating body composition is a strength, as BMI can mask unfavorable results. Z-scores for body composition results were calculated using a recent European reference population [29]. Furthermore, we also compared results with a group of healthy Norwegian children, which is considered a strength. However, we acknowledge that they might be healthier than the general population, which is a potential limitation of the results. Our small study included a heterogeneous patient group, reflecting the population of children undergoing HSCT. Hence, this limited the power of statistical analyses, and causal relationships could not be evaluated. A limitation of this study is the lack of data on physical activity, which is an important determinant of body composition and skeletal health [19]. In addition, DXA is an area measurement, so BMD may be underestimated in children with short stature or growth delay [28], even though skeletal age adjustment was applied in patients with available hand radiographs. Studies are required to establish clinically relevant cut-offs for body composition markers, such as ALMI, FMI, and FM%, in growing children and adolescents. More extensive studies of nutrition and its impact on health outcomes in pediatric HSCT are warranted.

## 5. Conclusions

Pediatric HSCT patients are at risk of a more unfavorable body composition with lower muscle mass, higher fat mass, and lower BMD during the first year post-transplant. Overall dietary intake was lower at 3 months, but improved by the 1 year mark, also with higher calcium and vitamin D intake. However, lower fiber and higher fat intake were concerning. Our results indicate a need for nutritional follow-up after transplantation, particularly targeting protein, fat, fiber, calcium, and vitamin D. The first year might represent a window of opportunity to prevent, address, and manage health challenges, ultimately promoting long-term musculoskeletal health and establishing a healthy lifestyle.

## Figures and Tables

**Table 1:** Patient characteristics at hospital admission, at 3 months and 1 year post-transplant, and in healthy children.

	HSCT Baseline *N* = 28		HSCT 3 Months *N* = 26		HSCT 1 Year *N* = 24		Healthy *N* = 50		*p*-Value Baseline vs. Healthy	*p*-Value 3 Months vs. Healthy	*p*-Value 1 Year vs. Healthy
Age, years, mean (SD)	10.3	(4.0)	10.3	(4.0)	10.9	(4.0)	10.0	(3.6)	0.764 ^a^		
Male, *n* (%)	19	(68)					18	(36)	0.014 ^b^		
Caucasian ethnicity, *n* (%)	24	(86)					49	(98)			
Anthropometrics											
- Weight, kg, mean (SD)	37.7	(18.9)	36.1	(17.4)	37.8	(18.1)	36.7	(14.4)	0.794 ^a^		
- Height, cm, mean (SD)	139.1	(26.5)	138.5	(26.5)	140.0	(24.0)	139.9	(18.4)	0.892 ^a^		
- Weight Z-score, mean (SD)	−0.33	(1.22)	−0.52	(1.1)	−0.58	(1.09)	0.06	(0.93)	0.113 ^a^	0.018 ^a^	0.011 ^a^
- Height Z-score, mean (SD)	−0.68	(1.23)	−0.77	(1.22)	−0.95	(1.08)	0.07	(1.18)	0.011 ^a^	<0.005 ^a^	<0.001 ^a^
- BMI Z-score, mean (SD)	0.10	(1.2)	−0.04	(1.0)	−0.04	(0.98)	0.02	(1.07)	0.738 ^a^	0.829 ^a^	0.843 ^a^
- Stunted, *n* (%)	5	(18)	5	(19)	5	(21)	2	(4)	0.091 ^b^		
- Thinness, *n* (%)	0	0	0	0	0	0	2	(4)			
- Normal BMI, *n* (%)	21	(75)	24	(93)	20	(83)	36	(72)			
- Overweight, *n* (%)	7	(25)	2	(7)	4	(17)	12	(24)	0.805 ^b^		
Diagnosis, *n* (%)											
- Malignant disease	18	(64)									
- Non-malignant disease	10	(36)									
Conditioning, *n* (%)											
- Myeloablative	24	(86)									
- TBI	7	(25)									
- Reduced intensity	4	(14)									
Donor type, *n* (%)											
- Matched unrelated donor	20	(71)									
- Matched related donor	7	(25)									
- Haploidentical donor	1	(4)									
GvHD, *n* (%)					7	(26)					
- Acute GvHD grade II-IV					7	(26)					
- Chronic GvHD					4	(15)					
Mortality, *n* (%)					4	(14)					

Abbreviations: BMI = body mass index (kg/m^2^), GvHD = graft-versus-host disease, HSCT = hematopoietic stem cell transplantation, SD = standard deviation. ^a^ Independent samples *t*-test, ^b^ Chi-square/Fisher’s Exact Test. Stunted height Z-score < −2, thinness BMI Z-score < −2, normal BMI Z-score −2 to 1, overweight (including obesity) BMI Z-score > 1.

**Table 2:** Daily provision (median, IQR) of energy, macronutrients, and micronutrients in patients at 3-month and 1-year post-transplant follow-up and in healthy children.

	HSCT Patients 3 Months *N* = 23		HSCT Patients 1 Year *N* = 18		Healthy Children *N* = 50		*p*-Value ^a^ 3 Months vs. 1 Year	*p*-Value ^b^ 3 Months vs. Healthy	*p*-Value ^b^ 1 Year vs. Healthy
	Median	IQR	Median	IQR	Median	IQR			
Kilojoule	5481	(4528–7458)	7691	(6128–8996)	8002	(6271–8585)	0.002	0.002	0.813
Kcal	1310	(1082–1783)	1838	(1465–2150)	1913	(1499–2052)	0.002	0.002	0.813
- Kcal/kg	49	(32–62)	52	(41–62)	52	(39–69)	0.009	0.200	0.911
Protein, g	42.4	(29.8–59.3)	62.6	(53.8–95.8)	72.5	(57.3–90.5)	0.002	<0.001	0.564
- Protein, g/kg	1.5	(1.3–1.8)	1.9	(1.6–2.4)	2.3	(1.8–2.7)	0.002	<0.001	0.196
Fat, g	55.5	(41.6–73.0)	78.5	(63.9–100.1)	67.0	(53.9–78.5)	<0.001	0.057	0.031
Carbohydrates, g	166.4	(128.0–249.1)	202.3	(175.2–234.2)	216.1	(174.7–249.8)	0.035	0.042	0.436
Fiber, g	7.9	(4.9–12.6)	14.6	(12.2–18.0)	18.3	(14.2–21.7)	<0.001	<0.001	0.006
- g/MJ	1.5	(0.8–1.9)	1.9	(1.6–2.2)	2.4	(2.1–2.7)	0.015	<0.001	0.005
Sugar, g	23.5	(5.9–60.7)	42.3	(26.0–80.3)	38.2	(27.2–53.4)	0.570	0.219	0.445
Calcium, mg ^c^	583	(480–876)	1062	(712–1322)	801	(652–985)	0.009	0.033	0.042
- % of RI	64	(46–110)	105	(88–119)	93	(67–117)	0.030	0.057	0.104
- Below RI (%)	53	(12)	39	(7)	54	(27)	0.095 ^d^	0.493 ^d^	0.272 ^d^
Vitamin D, µg ^e^	18.1	(11.9–28.7)	25.0	(16.0–30.9)	5.3	(2.7–9.9)	0.267	<0.001	<0.001
- % of RI	181	(119–287)	250	(160–309)	53	(27–99)			
- Below RI (%)	4	(17)	2	(11)	38	(76)	0.405 ^d^	<0.001 ^d^	<0.001 ^d^
Energy-%									
- Protein	11.8	(10.5–14.8)	14.9	(13.2–16.7)	16.3	(14.6–18.7)	0.028	<0.001	0.085
- Fat	33.9	(30.8–41.3)	37.6	(35.4–41.9)	33.2	(30.6–35.5)	0.084	0.196	0.001
- Carbohydrates	51.2	(42.1–54.4)	46.1	(42.5–51.2)	47.2	(45.2–52.2)	0.031	0.249	0.249
- Sugar	7.3	(1.9–16.5)	9.7	(5.9–15.2)	8.6	(6.3–11.0)	0.438	0.486	0.436

Abbreviations: IQR = interquartile range, MJ = megajoule, RI = recommended daily intake from Nordic Nutrition Recommendations 2023 [33]. ^a^ Wilcoxon signed-rank test, ^b^ Mann–Whitney U Test, ^c^ At 3 months, *n* = 19, ^d^ Chi-Square. ^e^ Vitamin D intake [22]. Four patients received supplementary parenteral nutrition and were not included in the analyses of calcium intake.

## Data Availability

The original contributions presented in this study are included in the article/Appendix A. Further inquiries can be directed to the corresponding author.

## Abbreviations

The following abbreviations are used in this manuscript:

ALM	Appendicular lean mass
ALMI	Appendicular lean mass index
BMD	Bone mineral density
BMI	Body mass index
DXA	Dual-energy X-ray absorptiometry
FM	Fat mass
FMI	Fat mass index
FM%	Fat mass percentage
GVHD	Graft-versus-host disease
HSCT	Hematopoietic stem cell transplantation
IQR	Interquartile range (25–75 percentile)
RI	Recommended daily intake
SD	Standard deviation
TBI	Total body irradiation
TBLH	Total body less head

## Appendix A

**Table A1:** Body composition and bone mineral density in HSCT patients and healthy children.

	HSCT 3 Months *N* = 18 ^a^		HSCT 1 Year *N* = 15		Healthy *N* = 46 ^a^		*p*-Value 3 Months vs. 1 Year ^b^	*p*-Value 3 Months vs. Healthy ^c^	*p*-Value 1 Year vs. Healthy ^c^
	Median	(IQR)	Median	(IQR)	Median	(IQR)			
ALMI Z-score	−1.69	(−2.35–−1.11)	−1.34	(−1.75–−0.53)	0.40	(−0.05–0.83)	0.007	<0.001	<0.001
ALMI (kg/m^2^)	4.4	(3.8–4.8)	5.1	(4.4–5.6)	5.3	(4.6–6.2)	<0.001	<0.001	0.180
FMI Z-score	0.25	(−0.74–1.20)	0.34	(−0.21–1.25)	−0.33	(−1.65–0.22)	0.278	0.027	0.012
FMI (kg/m^2^)	5.8	(3.8–8.6)	5.9	(4.7–9.1)	4.8	(3.3–5.6)	0.865	0.060	0.048
FM% Z-score	0.53	(−0.47–1.31)	0.59	(−0.06–1.49)	−0.98	(1.95–−0.28)	0.009	<0.001	<0.001
FM%	31.5	(26.1–40.4)	31.0	(25.6–41.6)	24.9	(21.1–27.7)	0.002	0.004	0.010
BMD TBLH Z-score	−0.8	(−1.4–−0.2)	−1.0	(−1.4–0.5)	0.3	(0.0–0.8)	0.107	<0.001	0.006
BMD L1–L4 Z-score	−0.5	(−1.6–0.1)	0.1	(−0.9–0.9)	0.2	(−0.3–0.8)	0.001	0.008	0.435

Abbreviations: ALMI = appendicular lean mass index, BMD = bone mineral density, FM% = fat mass percentage, FMI = fat mass index, HSCT = hematopoietic stem cell transplantation, IQR = interquartile range, L1–L4 = spine lumbar vertebrae L1–L4, TBLH = total body less head. ^a^ *n* = 16 at 3 months and *n* = 42 for healthy for ALMI, FMI, and body fat percentage results, as references were not available for children aged 5–6 years, BMD L2–L4 Z-score: *n* = 44 for healthy children. ^b^ Wilcoxon signed-rank test, ^c^ Mann–Whitney U Test.

**Table A2:** Body composition and bone mineral density in HSCT patients compared to healthy children.

	HSCT 3 Months *N* = 18 ^a^		HSCT 1 Year *N* = 15		Healthy *N* = 46 ^a^		*p*-Value 3 Months vs. 1 Year ^b,c^	*p*-Value 3 Months vs. Healthy ^c^	*p*-Value 1 Year vs. Healthy ^c^
	*n*	(%)	*n*	(%)	*n*	(%)			
ALMI Z-score									
- Normal > −1	3	(19)	6	(40)	40	(95)			
- Decreased ≤ −1	13	(81)	9	(60)	2	(5)	0.077	<0.001	<0.001
- Low −1 to > −2	7	(44)	8	(53)	2	(5)			
- Very low ≤ −2	6	(38)	1	(7)	0	(0)			
FMI Z-score									
- Normal < 1	10	(63)	9	(60)	38	(90)			
- High ≥ 1	6	(37)	6	(40)	4	(9)	0.032	0.020	0.015
FM% Z-score									
- Normal < 1	11	(69)	10	(67)	40	(95)			
- High ≥ 1	5	(31)	5	(33)	2	(5)	0.001	0.014	0.011
BMD TBLH Z-score									
- Normal > −1	10	(56)	7	(47)	46	(100)			
- Decreased ≤ −1	8	(45)	8	(53)	0	(0)	0.001	<0.001	<0.001
- Low −1 to > −2	7	(39)	7	(47)	0	(0)			
- Very low ≤ −2	1	(6)	1	(6)	0	(0)			
BMD L1–L4 Z-score									
- Normal > −1	11	(61)	12	(80)	35	(95)			
- Decreased ≤ −1	7	(39)	3	(20)	2	(5)	0.044	0.004	0.137
- Low −1 to > −2	5	(28)	3	(20)	2	(5)			
- Very low ≤ −2	2	(11)	0	(0)	0	(0)			

Abbreviations: ALMI = appendicular lean mass index, BMD = bone mineral density, FM% = fat mass percentage, FMI = fat mass index, HSCT = hematopoietic stem cell transplantation, L1–L4 = spine lumbar vertebrae L1–L4, TBLH = total body less head. ^a^ ALMI, FMI, and FM% were analyzed for *n* = 16 at 3 months and *n* = 42 for healthy, because the reference population was available for children aged ≥ 6 years, BMD L2–L4 Z-score: *n* = 44 for healthy children. ^b^ Thirteen HSCT patients were measured at both time points, ^c^ Chi-square/Fisher’s Exact Test.

**Table A3:** Age, energy, and macronutrient intakes (median, IQR) in patients receiving nutritional support compared to a regular diet at 3 months and 1 year after HSCT.

	3 Months After HSCT *N* = 23				1 Year After HSCT *N* = 18					
	Nutritional Support ^a^ *n* = 12		Regular Diet *n* = 11		Nutritional Support ^a^ *n* = 4		Regular Diet *n* = 14		*p*-Value ^b^	
	Median	IQR	Median	IQR	Median	IQR	Median	IQR	3-Months	1-Year
Age, years	7.0	(5.5–9.3)	12.7	(8.5–13.9)	6.7	(5.3–8.4)	13.3	(9.2–14.6)	0.023	0.026
Kilojoule	5727	(4707–7049)	5530	(4293–8265)	5962	(4933–6396)	8207	(7334–9195)	0.806	0.015
Kcal	1368	(1122–1682)	1310	(1022–1964)	1421	(1173–1524)	1955	(1750–2184)	0.902	0.015
- Kcal/kg	61	(47–68)	33	(28–49)	83	(61–85)	51.0	(40.7–53.9)	0.002	0.071
Protein, g	38.5	(26.9–57.3)	49.1	(31.3–59.3)	49.6	(40.0–59.2)	78.6	(57.5–102.4)	0.580	0.056
- Protein, g/kg	1.7	(1.5–1.9)	1.3	(0.9–1.5)	2.8	(2.0–3.3)	1.9	(1.5–2.2)	0.006	0.089
Fat, g	58.0	(43.8–73.7)	51.2	(36.3–67.5)	66.2	(49.4–88.7)	78.6	(63.9–100.1)	0.622	0.339
Carbohydrates, g	155.6	(133.2–221.5)	175.7	(120.9–265.8)	151.1	(128.4–175.5)	213.4	(189.9–237.0)	0.712	0.011
Fiber, g	6.4	(4.2–10.9)	9.4	(5.9–14.1)	12.4	(12.3–13.6)	16.0	(11.4–18.3)	0.281	0.457
- g/MJ	1.6	(0.7–1.9)	1.8	(1.1–1.9)	2.2	(2.1–2.6)	1.7	(1.6–2.1)	0.498	0.071
Energy-%										
- Protein	11.8	(10.5–14.1)	13.5	(11.5–15.1)	14.3	(11.9–16.6)	14.7	(13.4–18.6)	0.406	0.490
- Fat	34.8	(32.1–40.0)	31.8	(28.1–40.6)	41.2	(34.9–45.8)	36.4	(34.9–39.7)	0.185	0.288
- Carbohydrates	52.9	(46.9–54.1)	54.5	(41.7–58.5)	44.7	(39.7–51.3)	48.8	(44.4–53.9)	0.479	0.426

Abbreviation: HSCT = hematopoietic stem cell transplantation, IQR = interquartile range. ^a^ Nutritional support = oral nutrition support ± tube feeding ± parenteral nutrition. ^b^ Mann–Whitney U test between patients using nutritional support and a regular diet at 3-month and at 1-year follow-up.
